# Obesity Reduces Cognitive and Motor Functions across the Lifespan

**DOI:** 10.1155/2016/2473081

**Published:** 2016-01-12

**Authors:** Chuanming Wang, John S. Y. Chan, Lijie Ren, Jin H. Yan

**Affiliations:** ^1^Department of Neurology, The Affiliated Shenzhen Nanshan Hospital, Shenzhen University, Shenzhen 518000, China; ^2^Center for Brain Disorders and Cognitive Neuroscience, Shenzhen University, Shenzhen 518060, China; ^3^Department of Psychology, The Chinese University of Hong Kong, Shatin, Hong Kong; ^4^Department of Neurology, Shenzhen Second People's Hospital, Shenzhen University, Shenzhen 518035, China

## Abstract

Due to a sedentary lifestyle, more and more people are becoming obese nowadays. In addition to health-related problems, obesity can also impair cognition and motor performance. Previous results have shown that obesity mainly affects cognition and motor behaviors through altering brain functions and musculoskeletal system, respectively. Many factors, such as insulin/leptin dysregulation and inflammation, mediate the effect of obesity and cognition and motor behaviors. Substantial evidence has suggested exercise to be an effective way to improve obesity and related cognitive and motor dysfunctions. This paper aims to discuss the association of obesity with cognition and motor behaviors and its underlying mechanisms. Following this, mechanisms of exercise to improve obesity-related dysfunctions are described. Finally, implications and future research direction are raised.

## 1. Introduction

Obesity is the overaccumulation of fat which has aversive effects on health. The World Health Organization (WHO) defines overweight and obesity as body mass index (BMI) ≥ 25 and BMI ≥ 30, respectively [1]. Around the world, obesity has become a worrying health and social issue, threatening lives of thousands of people. According to the WHO [1], over 1.9 billion adults (39% adults) were overweight among which more than 600 million (13% adults) were obese. Childhood obesity is also common that 42 million children were overweight or obese in 2013 [1]. Considering its high prevalence, it is pressing to study the pathogenesis, manifestations, and prevention of obesity.

Obesity is related to a range of health-related problems, such as diabetes, heart disease, hypertension, and cancer [2]. Compared to normal-weight individuals, obese individuals have a reduced life expectancy [3]. Obese children show greater cardiovascular risk factors and persistence of obesity into their adulthood, which may be associated with higher likelihood of premature mortality [4, 5]. In addition to health problems, obesity is associated with poorer cognition and motor control, and altered brain plasticity. In this review, we first look into the behavioral manifestations of obese individuals' cognition and motor control capabilities. Next, obesity-related changes in brain plasticity will be discussed. Following this, the effects of physical exercise to combat obesity and obesity-related deficits in cognition and motor control will also be described. Finally, implications and future research directions are raised.

## 2. Cognition

Overweight and obesity are usually related to poorer cognition across lifespan [6–8]; however, the association between BMI and cognitive function is weaker in old age [9, 10], partly due to inaccurate adiposity measurement in the aged people [11]. Indirect evidence has shown an association between western high fat diet and impaired cognitive functions [12]. Based on BMI data, individuals who are overweight or obese fall in the lowest quartile of global cognition, verbal fluency, delayed recall, immediate logical memory, and intelligence [13].

Other than BMI, other adiposity measures are also related to cognitive performance and brain changes. Visceral adiposity is inversely correlated with verbal memory and attention. High visceral adiposity is associated with smaller hippocampus and larger ventricular volume [14]. There is also a negative correlation between waist-to-hip ratio and hippocampal volume and a positive correlation between waist-to-hip ratio and white matter hyperintensities [15]. Compared to BMI, central adiposity has a stronger association with the risk of developing cognitive impairment and dementia in women [16]. Hence, studies using BMI as the only indicator of obesity may not be sensitive enough to capture obesity-induced cognitive dysfunctions.

Neuroimaging studies demonstrate atrophy in the frontal lobes, anterior cingulate gyrus, hippocampus, and thalamus in older obese individuals [17]. BMI increase is associated with lower metabolic activity in the prefrontal cortex and cingulate gyrus, smaller gray matter volume in many brain regions (particularly prefrontal cortex), and deficient white matter integrity in the uncinated fasciculus which is a structure connecting the frontal and temporal lobes [18–22]. Smaller gray matter volume in the left orbitofrontal region is related to poorer executive performance in obese women [21].

Childhood obesity is related to the reduced executive function, attention, mental rotation, mathematics, and reading achievement [23–25]. Obese adolescents have deficits in a range of cognitive functions, such as attention and executive functions [26, 27]. An animal study shows that high fat diet induces similar morphometric and metabolic changes in juvenile and adult mice; however, only early exposure to high fat diet hurts relational memory flexibility and decreases neurogenesis [28]. Thus, early exposure to high fat diet may be particularly deleterious to cognition.

People with higher midlife BMI have lower global cognition than their thinner counterparts [29] and midlife obesity is related to the accelerated cognitive aging, but this association is weaker in older adulthood [30]. Both age and BMI contribute independently to decreased brain volume in middle and older adulthood [31]. It is more likely for an older adult to have lower cognitive abilities if he/she was overweight or obese during middle age [32, 33]. Midlife obesity is related to an increased pace of deterioration in executive functions and an increase in waist-to-hip ratio is associated with substantial reduction in total brain volume [34]. Lower BMI and waist circumference and higher fat-free mass are associated with slower cognitive decline [35]. Midlife overweight/obesity, particularly with metabolic abnormality, is associated with higher dementia risk in older adulthood [33, 36–40]. Moreover, high midlife BMI is related to neuron and myelin abnormalities [41]. Hence, midlife is a critical period in which the overweight/obese status can predict one's cognitive functions and brain health in later life [42].

## 3. Motor Control

Besides cognition, obesity also affects motor control capabilities, degrading daily functions and health [43]. Children who are obese or overweight are poorer in gross and fine motor control and have delayed motor development [44–50]. Obese boys have poorer motor skills and a reduced activity of daily living [51]. Obese girls of 6th and 7th grades participate in less physical activity and have lower enjoyment of physical activity [52]. Children with high BMI have lower level of run which is a fundamental motor skill based on which complex motor skills are learned [53]. Cliff et al. [54] observe that the prevalence of mastery of all fundamental motor skills is lower in overweight/obese children, especially for run, slide, hop, dribble, and kick. In addition to BMI, waist circumference is also related to children's and adolescents' ability to perform fundamental motor skills [55]. There is an inverse relationship of BMI with fine motor precision, balance, running speed and agility, and strength in the 1st graders [56]. Obese children also have difficulty in postural coordination and a heightened dependency on vision during locomotion which is rather automatic in nonobese children [57, 58].

Adiposity is related to muscle quality ratio that is associated with motor conduction velocity and finger tapping speed [59]. Obesity is related to greater fluctuation in handgrip force production [60]. Subcutaneous fatness can account for a significant variance of health-related and motor fitness [61]. Excessive fat mass is associated with poorer posture and walking [62]. In middle and older adults, a combination of high BMI (or waist circumference) and high blood pressure is related to lower motor speed and manual dexterity [63]. During postural control, obese individuals require greater attention resources to maintain balance during unipedal stance [64]; this implicates that obese people consume attention resources to compensate for their motor deficits.

## 4. Obesity-Related Changes in Brain Plasticity

A number of factors may mediate obesity's effects on cognition and motor behaviors. For example, obesity may affect brain structure, leptin and insulin dysregulation, oxidative stress, cerebrovascular function, blood-brain barrier, and inflammation [11, 65–71]. Some also suggest that obesity-related changes in metabolism interact with age to impair brain functions [72].

In terms of brain structure, obese individuals have lower cortical thickness in the left superior frontal and right medial orbitofrontal cortex. The volumes of ventral diencephalon and brainstem are also reduced in obese people [73]. There is also a negative relationship between neuronal injury and gray matter density in hippocampus and cerebellum in overweight and obese individuals [74]. It is suggested that the medial orbitofrontal cortex, hippocampus, and cerebellum are involved in reward-based learning, memory, and motor control and learning [75–77]; structural alterations in these regions may be associated with deficits in cognitive and motor domains. Hitherto, the mechanisms underlying obesity's effects on brain structure are not clear.

High fat diet increases oxidative stress and inflammatory signaling in the brain [78]. Diet-induced obesity promotes reactive oxygen species in the brain which is associated with both body weight and adiposity [79, 80]. In children, intake of saturated fatty acids impairs both relational and item memory [81]. Occurrence of 15-week obesity during childhood can induce permanent epigenetic changes in rat's brain [82]. In rats, triglycerides diminish the passage of insulin-like growth factors (IGFs) into the brain through cerebrospinal fluid, impair hippocampal long-term potentiation, and impede leptin transportation across the blood-brain barrier [83–85]. Juvenile exposure to high fat diet impairs long-term spatial memory, but not short-term memory, suggesting a selective impairment of consolidation which is likely contributed by increased proinflammatory cytokine expression in the hippocampus [86]. Moreover, consumption of western diet is thought to degrade blood-brain barrier, which consequently damages hippocampus and leads to dementia [87]. Relative to those having normal diet, mice consuming high fat diet for 17 days develop insulin resistance in cerebral cortex tissues, degraded synaptic integrity, and poorer spatial memory [88].

Leptin is a cytokine and satiety hormone helping regulate appetite and energy expenditure. It can cross the blood-brain barrier and binds to presynaptic GABAergic neurons to produce its effects [89, 90]. Leptin production is increased in obesity [91]. As leptin receptors are widespread in the brain (e.g., throughout the cortex and the hippocampus), leptin can modulate memory processes [92]. Obese mice with impaired leptin signaling have deficits of hippocampal-dependent memory [93] and increased basal hippocampal inflammation [94, 95]. Leptin is related to neurogenesis, axonal growth, and synaptogenesis in addition to hypothalamic functions [96–98]. For hippocampal neurons, leptin plays a role in long-term potentiation and depression and thus is important for synaptic plasticity [92, 99, 100]. Compared to those with low leptin level, the elderly with high leptin level show less cognitive decline during aging [101]. High leptin level in individuals with small waist circumference is related to less cognitive decline over 10 years [102]. The presence of leptin may decrease the production of amyloid and speed up the removal of *β*-amyloid [103]. Older adults with higher leptin level are at a lower risk of developing dementia [104]. Obese individuals usually develop leptin resistance [105] which results in an increase in food intake and alteration of energy expenditure [90].

The circulating levels of insulin and signaling pathway are altered in obesity; this interacts with inflammatory processes to modulate cognition and behaviors [106]. Insulin plays a role in modulating hippocampal synaptic plasticity [107]. As insulin receptors are widespread in hippocampal and cortical brain structures, insulin signaling can contribute to the formation of declarative memory [108]. Insulin concentrations vary with adiposity and there is a negative relationship between the amount of visceral fat and insulin sensitivity [109]. Insulin resistance can result from high fat consumption or obesity [110, 111]. Dysfunctional insulin signaling can induce inflammation and promote *β*-amyloid and tau pathology, contributing to neurodegeneration [112, 113]. Insulin resistance can mediate cognitive impairment and neurodegeneration as insulin and IGFs can regulate neuronal survival, metabolism, and brain plasticity [114, 115]. During insulin resistance, there is a failure of cells to metabolize glucose, which consequently triggers an increase of insulin level. Insulin signaling is related to tau phosphorylation, an early pathology of Alzheimer's disease [116, 117]; this is complementary to the fact that there are a large number of insulin-sensitive glucose transporters in the medial temporal lobe [118]. Thus, insulin dysregulation in the obese people likely confers a greater risk of dementia to them.

The adipose tissue produces many substances for metabolism (adipokines, such as BDNF) and inflammation (cytokines, such as leptin). Many cytokines, such as interleukin-1, produced by the adipose tissue can cross the blood-brain barrier and affect cognitive functions through neuroinflammation [95, 119]. Adiponectin is involved in regulating glucose level and fatty acid breakdown. Similar to leptin, it exerts its effects in the brain to bring about weight reduction [120]. Its level is negatively associated with adiposity and can protect hippocampal cells [119]. Reduced hippocampal adiponectin levels are observed in aging animals, independent of high fat diet intake [121]. Thus, adiponectin is important for neurodegeneration prevention.

Neurotrophins, such as IGF-1 and BDNF, can mediate obesity's effects on cognition and behaviors. IGF-1 is mainly produced in liver and binds to the IGF-1 or insulin receptors to exert its effects to stimulate cell growth and proliferation and promote *β*-amyloid clearance in the brain [122]. Obese individuals usually show IGF-1 resistance, degrading their capability to prevent *β*-amyloid deposition and neurodegeneration [114, 123]. Besides, BDNF can bind to many receptors, such as TrkB and LNGF receptors, to support neuronal survival and stimulate neurogenesis and synaptogenesis [124–126]. Cardiometabolic diseases are usually associated with low BDNF [127]. BDNF promotes neuronal differentiation and survival, neurogenesis, and brain plasticity and is thus particularly crucial for learning and memory [128]. High fat diet reduces BDNF level in the hippocampus [129], and the impaired hippocampal synaptic plasticity and cognition are possibly through BDNF's effects on dendritic spines [130]. Diet-induced obesity reduces hippocampal expression of BDNF and presynaptic synaptophysin, which are related to an impairment of spatial learning in mice [131].

Although mounting evidence shows that obesity is associated with structural and functional brain changes, the causal link between them requires further investigations. In contrast, the causal link between diet and brain changes is much clearer. The composition of gut microbiota appears to be causally related to obesity [132–134], playing a significant role in body weigh regulation since birth [135, 136]. Gut microbiota plays a key role in childhood obesity and brain development [137, 138]. A comparison of germ-free mice and conventionally reared mice has demonstrated that germ-free mice are leaner and more resistant to diet-induced obesity [139]. Obese and nonobese individuals have different diversity and composition of gut microbiota [140, 141]. As gut microbiota controls energy extraction and storage in the body, significant changes in gut microbiota can result in obesity and insulin resistance [139, 140, 142].

It has been suggested that diet can influence gut microbiota which in turn impacts the brain and behaviors through neural, hormonal, immune, and metabolic pathways [143, 144]. Transplantation of gut microbiota of diet-induced obese mice to lean mice is sufficient to bring about neurobehavioral changes through increasing neuroinflammation and disrupting cerebrovascular homeostasis [145, 146]. Mice consuming high energy diet containing higher percentage of* Clostridiales* and lower expression of* Bacteroidales* have poorer cognitive flexibility [147]. In humans, the* Firmicutes/Bacteroidetes* ratio is positively associated with BMI [148]. Gut microbiota can modulate a range of neurotrophins, such as BDNF and synaptophysin, to affect neural plasticity [149, 150]. Thus, diet changes gut microbiota which influences neurophysiology and neurotrophins, eventually impacting cognition and behaviors.

Previous results have shown that obesity-related brain plasticity alteration is a multifaceted issue, which can inflict permanent harm to individuals in their early ages. Thus, it would be optimal to combat obesity during childhood.

## 5. Exercise Improves Brain Functions

Exercise can improve physical and cognitive performance, and quality of life in the elderly [151–155]. In humans, those who are highly fit or aerobically trained have greater prefrontal and parietal activations for spatial selection and inhibitory functioning [156]. There is a positive relationship between aerobic fitness and spatial memory which is mediated by hippocampus volume [157]. Aerobic training can increase hippocampal volume of the elderly (with or without mild cognitive impairment) and increases plasma BDNF level in both patients of Alzheimer's disease and healthy controls [158–163]. Regular physical activity is related to better cognition, less cognitive decline, and a lower risk of developing dementia [164, 165]. As young as children, aerobic fitness can predict cognitive performance over time [166]. Besides cognition, higher level of physical activity is related to a reduced white matter hyperintensity burden on motor function in the aged people [167]. BDNF concentration is associated with retention performance of motor skill after learning [168]. Lifelong exercise can preserve white matter microstructure related to motor control and coordination in the elderly [169]. In addition, regular physical activity has long been suggested to be an effective way to improve obesity and related problems [170, 171]. Exercising 5 days per week for 15 weeks can improve executive functions in overweight children [172]. High-intensity physical activity (both aerobic and endurance training) for 4 months improves cognition and oxygen extraction in obese individuals [173].

The effectiveness of exercise may be moderated by exercise intensity and duration, and exerciser's developmental stage [174, 175]. Exercise intensity can be related both to behavioral outcomes and to changes in brain structure and BDNF level. High dose group improves planning more than the low dose group [172]. Greater amount of physical activity in early life is associated with larger prefrontal and hippocampal volumes [176]. Individuals receiving low-intensity exercise, but not high-intensity, show increased BDNF expression [177]. BDNF level depends on exercise intensity [178]; some observe that moderate-intensity exercise is the most effective to promote BDNF in the elderly [179]. Thus, it seems that a moderate intensity of exercise is optimal. In addition to exercise intensity, duration of exercise is also crucial. Tomporowski et al. [180] fail to observe any augmentation of task switching performance after a single bout of moderately intense exercise. In midlife mice, only 4-month (but not 2-month) running training can trigger activation of the antiamyloidogenic, prosurvival, synaptogenic, and neuroprotective pathways [181]. Wheel running for 14 days can increase cell proliferation in the dentate gyrus whereas wheel running for 56 days can additionally facilitate long-term potentiation in this region [182]. These show that a longer duration of exercise favors changes in the brains. Moreover, the developmental stage of exerciser is associated with benefits of exercise. Four-week exercise can improve recognition memory in adult rats, but no such enhancement can be recorded 2 weeks after cessation of training. However, in adolescent rats, the enhancement of recognition memory is preserved [183]. These nicely demonstrate that younger animals benefit more from exercise.

At the neuronal level, physical activity can enhance neurogenesis, neuroadaptation, and neuroprotection though the actions of neurotrophic factors [184–190]. Hippocampal function is restored by physical activity through enhancing the expression of neurotrophic factors to promote neurogenesis, angiogenesis, and synaptic plasticity [191–193]. For example, BDNF level increases with physical activity, particularly regular exercise [194, 195]. It is found that BDNF can stimulate DNA repair to protect cortical neurons against oxidative stress [196]. Short bout of mild exercise for 5 weeks improves both oxygen consumption and long-term spatial learning and memory in aged rats which is associated with hippocampal BDNF level [197]. Following physical activity, hippocampal BDNF level and TrkB receptor activation are increased [198]. The elevated BDNF level in the dentate gyrus is sufficient to induce spatial memory improvement [199]. A week of voluntary exercise is sufficient to increase the activity of tissue type plasminogen activator to facilitate the cleavage of proBDNF into mBNDF [200]. Also, exercise promotes sirtuin 1, stimulates mitochondrial biogenesis, and prevents neurodegeneration [201].

Exercise can be related to structural brain changes [202]. A 7-day exercise intervention can increase gray matter volumes in the motor, somatosensory, association, and visual cortices in rats [203]. Exercising for 6 months reduces default mode network activity in the precuneus [204] while one-year walking increases functional connectivity within the default mode network and frontal executive network [205]. Regular physical activity can reduce proinflammatory and increase anti-inflammatory signaling and reduce oxidative stress in aged animals [206, 207]. Exercise also reduces peripheral risk factors, such as diabetes and cardiovascular diseases which are associated with neurodegeneration [208]. Furthermore, vasculature is altered after exercise. In middle-aged rats, total length and surface area of cortical capillaries are increased after running [209]. Aerobic exercise at midlife can improve vascular dysfunctions, astrocyte hypertrophy, and myelin dysregulation associated with sedentary lifestyle [210, 211].

**Figure 1:**
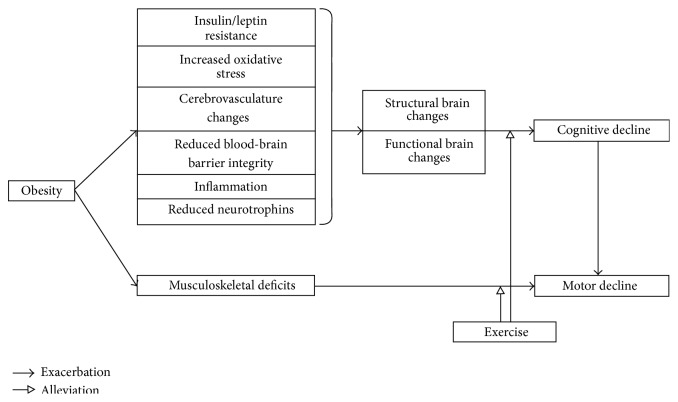
Factors mediating the effects of obesity and exercise on cognition and motor behaviors. Obesity affects cognition mainly through brain changes and influences motor behaviors through degrading the musculoskeletal system. Exercise can alleviate the deleterious effects of the obesity-related mediators on cognition and motor performance.

Exercise is associated with a range of improvements in the brain through a range of mechanisms in individuals of different weight statuses (Figure 1). The effectiveness of exercise depends on the training parameters, such as intensity, duration, and developmental stage of exerciser. Previous research results have consistently suggested that moderately intense exercise for a long enough period of time is especially beneficial for young exercisers.

## 6. Implications and Future Research

More and more people are becoming obese, producing aversive effects on their cognition, motor behaviors, and quality of life [1]. Previous research has suggested that altered brain structure and activation mediate obesity's influences on cognition [17–21], whereas obesity influences the musculoskeletal system to degrade motor performance [59]. As motor performance partly depends on cognitive ability [64], obesity may indirectly contribute to motor deficits through cognitive decline (Figure 1).

Substantial research has shown that obesity affects our cognition and motor behaviors through different mechanisms, possibly through altering brain structure, leptin/insulin regulation, oxidative stress, cerebrovascular function, blood-brain barrier, and inflammation [11, 65–71]. The validity of these proposed mechanisms requires further examinations.

Regular physical activity benefits both cognition and motor behaviors. It is suggested that moderately intense exercise for a long enough period of time seems favorable; however, the training parameter for optimal outcomes remains to be determined. Most of the previous research focuses on aerobic exercise; the efficacy of anaerobic exercise to improve obesity and related dysfunctions is not well understood. More efforts should be devoted to investigate the efficacy of anaerobic exercise, in comparison with aerobic exercise. Moreover, starting exercising in young age is particularly important to protect from neurodegeneration in old age. As childhood obesity is becoming more prevalent [23–25], introducing physical activity intervention in childhood may help children improve obesity and prevent age-related functional decline in old age.

In addition to exercise, leptin replacement therapy, inhaled insulin therapy, and caloric restriction have also been proposed to improve obesity. Leptin is responsible for energy balance and body weight and can affect neurogenesis and brain functions [212]. It enhances immune response and regulates inflammation [212]. It is observed that 18-month leptin replacement therapy increases gray matter concentration and activations in brain regions implicated in hunger and satiation neural circuits [213, 214]. During weight loss, leptin is reduced, facilitating food intake. Leptin therapy helps sustain weight loss [215].

There are insulin disturbances in obese individuals [216, 217]. Insulin resistance plays an important role in obesity and cognitive impairments [218]. It is found that intranasal insulin exerts anorexic effects, promoting satiety and regulating food intake [219, 220]. Inhaled insulin reaches the brain through olfactory nerves and specific receptors in blood-brain barrier to exert its effects [221]. Caloric restriction also improves obesity and reverses deficits in leptin receptor protein and signaling associated with diet-induced obesity [222]. After 3 months of caloric restriction, serum BDNF increases in overweight and obese individuals [223]. Diet-induced weight loss is related to a decrease in plasma free fatty acid and improvement in episodic memory [224]. Hitherto, the efficacy of leptin replacement therapy, inhaled insulin therapy, and caloric restriction on cognition and motor behaviors is poorly understood, which warrants further verification.

## 7. Conclusions

Obesity has become a worrying health and social issue. It affects cognition mainly through altering the brain structures and functions [17–21], and motor performance through degrading musculoskeletal system [59]. Obesity can affect brain structure, leptin/insulin dysregulation, oxidative stress, cerebrovascular function, blood-brain barrier, and inflammation [11, 65–71], which are involved in the deterioration of cognitive and motor functions. A host of previous research has suggested that exercise can improve both obesity-related cognitive and motor declines. As more and more people develop obesity in young age, introducing exercise intervention early would result in the greatest benefits.
